# Identification of VCAN as Hub Gene for Diabetic Kidney Disease Immune Injury Using Integrated Bioinformatics Analysis

**DOI:** 10.3389/fphys.2021.651690

**Published:** 2021-09-07

**Authors:** Qiannan Xu, Binjue Li, Yucheng Wang, Cuili Wang, Shi Feng, Lu Xue, Jianghua Chen, Hong Jiang

**Affiliations:** ^1^Kidney Disease Center, The First Affiliated Hospital, College of Medicine, Zhejiang University, Hangzhou, China; ^2^Key Laboratory of Nephropathy, Hangzhou, China; ^3^Institute of Nephropathy, Zhejiang University, Hangzhou, China; ^4^Department of Otolaryngology Head and Neck Surgery, Shanghai Ninth People’s Hospital, Shanghai Jiao Tong University School of Medicine, Shanghai, China; ^5^Ear Institute, Shanghai Jiao Tong University School of Medicine, Shanghai, China; ^6^Shanghai Key Laboratory of Translational Medicine on Ear and Nose Diseases, Shanghai, China

**Keywords:** bioinformatics analysis, VCAN, tubulointerstium, immune injury, diabetic kidney disease

## Abstract

**Background:** Diabetic kidney disease (DKD) is a leading cause of chronic kidney disease in China. Tubular injury contributes to the progression of DKD. Our study was conducted to explore the differential gene expression profiles between kidneys from patients with DKD and kidney living donors (LDs).

**Methods:** In total, seven DKD and eighteen LD gene expression profiles from the GSE104954 dataset were downloaded from the Gene Expression Omnibus database. Differentially expressed genes (DEGs) were analyzed in R with the limma package. DEGs were uploaded to the g:Profiler online database to explore the Gene Ontology (GO) and Kyoto Encyclopedia of Genes and Genomes (KEGG) pathways. Ingenuity pathway analysis (IPA) was carried out using online IPA software. Weighted gene co-expression network analysis (WGCNA) was performed using the WGCNA R package. By integrating DEGs and genes from the top 1 phenotype-gene associated module, we determined the hub gene. We next tested the hub gene, VCAN, in the GSE30122 dataset. We also validated the versican levels in human kidney tissues, explored immune cell type enrichment using an online database xCell, and investigated the correlation between cell types and VCAN expression.

**Results:** A total of 563 DEGs was identified. A large number of pathways were involved in the immune response process according to the results of GO, KEGG, and IPA. Using WGCNA, we selected the lightcyan module in which genes showed the strongest correlation with the phenotype and smallest *P*-value. We also identified VCAN as a hub gene by integrating DEG analysis and WGCNA. Versican expression was upregulated in human diabetic kidney tissue. Moreover, versican was speculated to play a role in immune injury according to the enrichment of functions and signaling pathways. VCAN transcript levels correlate with the assembly of immune cells in the kidney.

**Conclusion:** Immune processes played an essential role in DKD tubulointerstitium injury. The hub gene VCAN contributed to this process.

## Introduction

Diabetic kidney disease (DKD) has been a leading cause of chronic kidney disease (CKD) since 2011, surpassing glomerulonephritis in China (Zhang et al., 2016). Diabetes accounts for 30–50 % of all CKD cases and affects 285 million (6.4%) adults worldwide (Webster et al., 2017). Furthermore, the number of patients suffering from DKD has been increasing.

Given the mortality and morbidity of DKD, numerous studies have sought to determine the pathogenesis of DKD and promote pharmaceutical development aiming to slow and even reverse the progression of DKD. It is widely acknowledged that hemodynamic alterations, metabolic derangement, immune dysregulation, and filtration barrier damage cause kidney damage in DKD (Bonner et al., 2020). Furthermore, increasing evidence has shown that the renal tubulointerstitium acts as an initiator and major determinant of DKD pathogenesis (Nath, 1992; Tang et al., 2011; Tang and Lai, 2012; Gilbert, 2017; Zeni et al., 2017). As a consequence of decreased O_2_ delivery, mitochondrial dysfunction, increased O_2_ consumption, and non-ischemic pathways, renal tubule cells and the interstitium are damaged by apoptosis and fibrosis (Gilbert, 2017). Accordingly, some clinical trials, including the use of ARB/ACEI (IDNT (Rodby et al., 2000), RENAAL (Brenner et al., 2001)), SGLT-2 inhibitor (EMPAREG (Zinman et al., 2015), CANVAS (Neal et al., 2017), and CREDENCE (Perkovic et al., 2019), and GLP-1 analogs LEADER (Marso et al., 2016), REWIND (Gerstein et al., 2019) have demonstrated the effectiveness of slowing the progression of DKD. The role of inflammation in DKD progression has received wide attention in recent years (Yang and Mou, 2017; Hickey and Martin, 2018; Matoba et al., 2019; Tang and Yiu, 2020). However, no major clinical trials have targeted immune disorders in DKD because the underlying mechanisms are not well-understood.

Currently, the development of high-throughput technologies and online bioinformatics databases has enabled researchers to explore disease-related genes and the underlying mechanisms of diseases. As a widely used bioinformatics analysis method, weighted gene co-expression network analysis (WGCNA) clustered genes with similar expression patterns and provided trait-related gene information relying on expression data values. Although many studies have focused on exploring the genome expression of DKD, none has focused on the tubulointerstitium of DKD samples, and most bioinformatics studies lacked verification.

In this study, we selected a subset of microarray gene expression profiles from GSE104954, which was uploaded to the Gene Expression Omnibus database in a previous study (Grayson et al., 2018). Using WGCNA-based methods, we first identified VCAN as a hub gene with an essential role in the immune response during DKD progression. We then validated that versican was differentially expressed in kidney tissues from patients with DKD and in kidney living donors (LDs).,

## Materials and Methods

### Data Download and Preprocessing

The microarray gene expression profiles of renal tubulointerstitium were downloaded from the Gene Expression Omnibus database^1^. Data from GSE104954, including the data of seven patients with DKD (GSM2811029-GSM2811035) and eighteen LDs (GSM2811043-GSM2811060), were used as query arrays. Data from GSE30122 were used for validation. Raw data were downloaded, and analysis was conducted using the affy package in R version 3.6.2. Before being included in the analysis, the data were evaluated by the normalized unscaled standard error (NUSE), RNA degradation, relative log expression (RLE), probe level models (PLM), principal component analysis (PCA), and sample clustering, and, finally, the GSM20811043 was discarded.

### Identification of Differentially Expressed Genes (DEGs)

DEGs were analyzed using the limma package. The cutoff criteria of DEGs were as follows: adjusted *P*-value < 0.05 and |log_2_ fold-change| > 1. Volcano plots and heatmaps were created using the ggplot2 package in R.

### Function Analysis

An online database, g:Profiler^2^, was used for functional profiling of the DEGs. The top 10 ranked from four sub-databases, including molecular function, cellular component, biological process, and KEGG, were demonstrated separately in bubble plots using the ggplot2 package. Ingenuity Pathway Analysis (IPA) software (Qiagen, Hilden, Germany) was also used to explore the pathways involved. The top 30 canonical pathways and top network were determined.

### WGCNA

Gene expression valuation and hierarchical cluster analysis were carried out using the WGCNA R package (version 1.61^3^). Gene expression array from GSE104954 was used for analysis. The main processes used in WGCNA were co-expression network construction and module identification, identification of disease-associated modules, and enrichment analysis of key modules. Module- and phenotype-associated genes were screened under the following conditions: gene significance (GS) > 0.2 and module membership (MM) > 0.8. These genes were further imported into Cytoscape 3.7.2 (Shannon et al., 2003) to identify the hub genes. Hub genes were ranked using cytohubba (Chin et al., 2014) by several topological algorithms including degree, edge percolated component, maximum neighborhood component, density of maximum neighborhood component, maximal clique centrality, and centralities based on shortest paths, such as bottleneck, eccentricity, closeness, radiality, betweenness, and stress. Subsequently, hub genes intersected with the DEGs.

### Validation of Hub Gene

GSE30122 was utilized as a validation array to further verify the hub gene. Paraffin-embedded kidney sections were collected from 6 healthy living transplant doners and 6 DKD patients who were diagnosed with pathology in 2021. The experiment protocols were approved by the Research Ethics Committee of The First Affiliated Hospital, College of Medicine, Zhejiang University. Kidney paraffin sections from patients with DKD and LDs were stained with versican. Briefly, paraffin-embedded sections were dewaxed, incubated in citrate buffer at 95–98°C for 10 min for antigen retrieval, followed by in 0.3% H_2_O_2_ for 30 min at room temperature to block endogenous peroxidase in blocking buffer (5% bovine serum albumin) for 30 min to block non-specific binding, and then incubated with anti-versican antibody (Abcam, Cambridge, United Kingdom, ab19345, 1:150) overnight at 4°C, followed by incubation with secondary antibody for 30 min at room temperature. The DAB substrate solution was applied to visualize the color of primary antibody staining. The sections were counterstained with hematoxylin, dehydrated, vitrified, and sealed with neutral balsam.

### Gene Function Prediction

Gene expression data were uploaded to the xCell database^4^ for cell type enrichment analysis. Furthermore, correlation analysis between the VCAN gene and cell types was conducted using SPSS software (version 23; SPSS, Inc., Chicago, IL, United States).

## Results

### Identification of DEGs

**FIGURE 1:**
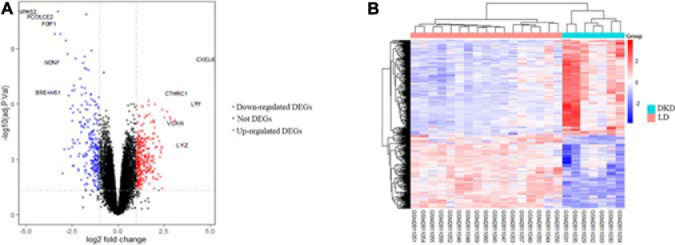
DEGs between seven patients with DKD and seventeen LDs from GSE104954. **(A)** Volcano plot of all genes in GSE104954 and top 10 DEGs were annotated. **(B)** Heatmap of DEGs identified.

After integrating quality evaluation of all transcriptome data from DKD and LD samples (Supplementary Figure 1), we omitted GSM2811043. Therefore, kidney transcriptome data from seven patients with DKD and seventeen LDs were included for further analysis. We identified 563 DEGs between the DKD and LD groups. These DEGs were defined based on adjusted *P*-values < 0.05 and log_2_ fold-change > 1. Among the DEGs, 316 genes were upregulated, and 247 genes were downregulated. All genes are displayed in Figure 1, and DEGs are listed in Supplementary Table 1. The top 30 DEGs ranked in order of log_2_ fold-change are listed in Table 1. Additionally, the top 10 ranked in order of log_2_ fold-change were annotated in a volcano plot.

**TABLE 1:** Top 30 DEGs identified in gene expression microarray analysis of seven patients with DKD and seventeen LDs.

Gene	Log_2_ fold-change	*P*-value	Adj. *P*-Value	Change
NPHS2	−4.90159	5.06E−16	9.84E−12	Downregulated
CXCL6	4.751296	2.16E−12	3.60E−09	Upregulated
LTF	4.244363	1.88E−09	1.02E−06	Upregulated
PCOLCE2	−4.22857	3.20E−15	1.73E−11	Downregulated
BRE-AS1	−3.7554	2.91E−10	2.34E−07	Downregulated
FGF1	−3.73582	9.72E−15	4.21E−11	Downregulated
NDNF	−3.55415	3.42E−12	5.28E−09	Downregulated
LYZ	−3.499337	2.79E−06	0.000161	Upregulated
USP2	−3.46439	2.86E−10	2.34E−07	Downregulated
CLIC5	−3.4096	5.40E−14	1.68E−10	Downregulated
PDK4	−3.25144	9.09E−16	9.84E−12	Downregulated
CTHRC1	3.165899	3.56E−10	2.70E−07	Upregulated
DUSP1	−3.1202	5.43E−14	1.68E−10	Downregulated
VCAN	3.11024	5.16E−08	1.04E−05	Upregulated
CPA3	3.090068	3.49E−08	8.05E−06	Upregulated
ALB	−2.99175	0.000588	0.005989	Downregulated
PSPH	−2.91351	6.92E−06	0.000289	Downregulated
FCER1A	2.848281	2.97E−08	7.15E−06	Upregulated
TAC1	2.838673	2.03E−08	5.49E−06	Upregulated
ST6GALNAC3	−2.82394	1.41E−13	3.81E−10	Downregulated
PODXL	−2.72476	9.63E−13	2.09E−09	Downregulated
SIK1	−2.66836	5.81E−10	3.82E−07	Downregulated
CYP27B1	−2.6602	6.25E−06	0.000271	Downregulated
ALOX5	2.658193	8.53E−09	2.80E−06	Upregulated
ZBTB16	−2.58168	2.69E−05	0.000708	Downregulated
ERRFI1	−2.56073	4.99E−09	1.86E−06	Downregulated
CX3CR1	2.508004	2.22E−08	5.80E−06	Upregulated
SOST	−2.48456	4.88E−06	0.000229	Downregulated
IP6K3	−2.48175	3.85E−08	8.51E−06	Downregulated
WT1	−2.47762	2.33E−10	2.02E−07	Downregulated

### Functional Enrichment Analysis of DEGs

**FIGURE 2:**
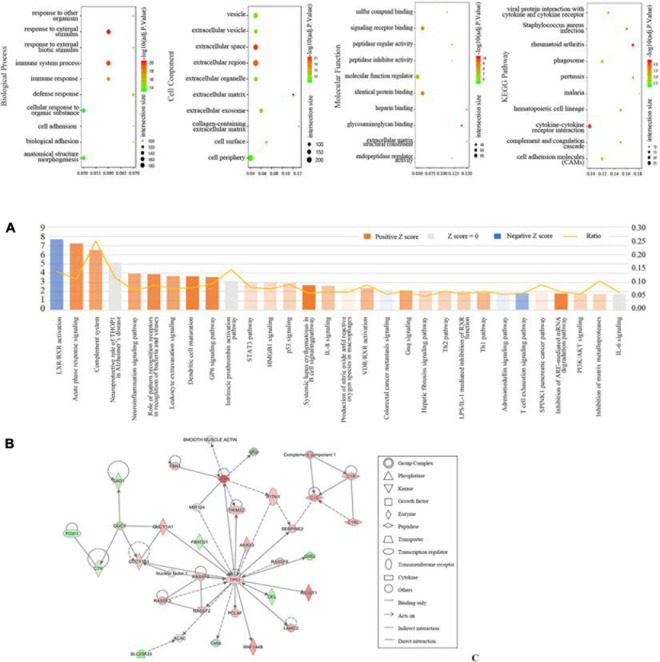
Function profiling of DEGs between seven patients with DKD and seventeen LDs from GSE104954. **(A)** Bobble plots of GO and KEGG. **(B)** Top 30 canonical pathways according to IPA. **(C)** Top 1 network according to IPA.

DEGs were uploaded to g:Profiler to identify GO and KEGG. As shown in Figure 2A, the most involved process or component in GO included immune system process, response to an external stimulus, immune response (biological process), external space, external region (cell component), glycosaminoglycan binding, signaling receptor binding, and identical protein binding (molecular function). The significantly enriched KEGG pathways were involved in rheumatoid arthritis, cytokine-cytokine receptor interaction, and *Staphylococcus aureus* infection. Additionally, DEGs were analyzed using IPA software. The canonical pathways were enriched in various aspects, including LXR/RXR activation, acute phase response signaling, and the complement system (Figure 2B). We also determined the top network involved in connective tissue disorders, dermatological diseases and conditions, and developmental disorder (Figure 2C).

### Construction of Weighted Co-expressed Network and Identification of Trait-Related Module

**FIGURE 3:**
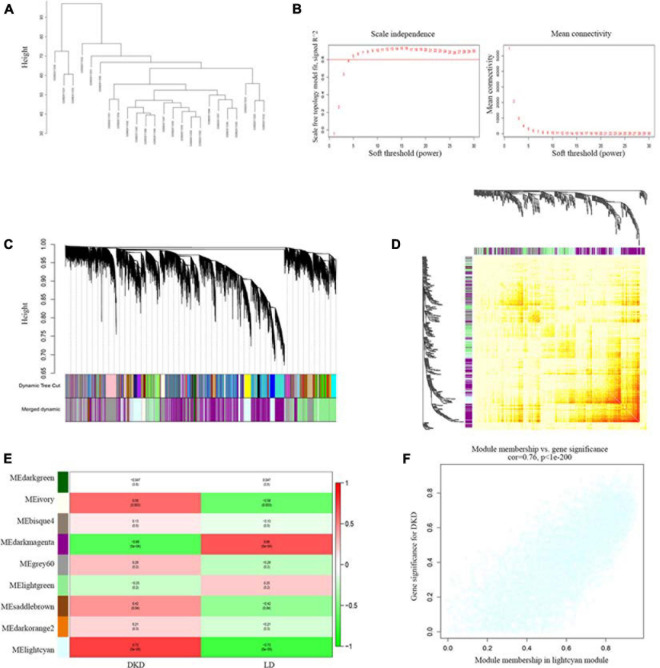
WGCNA of seven patients with DKD and seventeen LDs gene expression profiles from GSE104954. **(A)** Sample clustering based on co-expressed data enrolled. **(B)** Analysis of soft-thresholding powers *(fi)* to fit the scale-free topology model and mean connectivity of the soft-thresholding powers. With a scale-free topological criterion >0.8, 6 was chosen as the most fit power value. **(C)** Dendrogram of the gene modules. The branches represent different gene modules, and each leaf represents a gene in the cluster dendrogram. **(D)** Heatmap of the weighted gene co-expression correlations of all genes. **(E)** Correlation between module eigengenes and clinical traits. The clinical traits include DKD and LD. The corresponding correlations and *P*-values were presented. **(F)** Lightcyan module was identified to have the highest positive correlation with DKD-related clinical traits (correlation index = 0.76, *P* < 1E–200).

One samples were discarded (GSM2811043) (Supplementary Figure 1), the left 7 DKD and LD kidney samples were clustered (Figure 3A). We chose a power value of 6 as the closest value of the scale-free topological fit index of 0.8 (Figure 3B). We analyzed the correlation between genes and modules and between different genes (Figures 3C,D) and then determined the relationship between modules and traits (Figure 3E). The lightcyan module was the most positively correlated with DKD (Pearson correlation ratio, 0.73) with the lowest *P*-value (5e−5). Furthermore, genes in the lightcyan module showed a strong correlation with module membership (Figure 3F).

### Identification of Hub Genes

**FIGURE 4:**
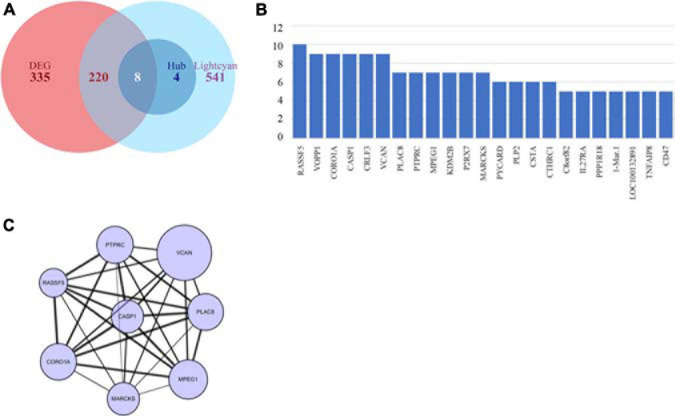
Identification of key genes by intersecting DEGs and hub genes. **(A)** Venn plot of DEGs and hub genes. **(B)** Hub genes identified using cyto-hubba. **(C)** Interaction of intersect genes using cytoscape.

In the lightcyan module, we used GS > 0.2 and MM > 0.8 as cutoffs. We acquired a set of 773 module member significantly related genes. These genes were analyzed in Cytoscape by using cytohubba to explore hub genes (Figure 4B). After integrating the results of WGCNA and DEGs, we identified 8 hub genes, namely *VCAN*, *PTPRC*, *RASSF5*, *CASP1*, *PLAC8*, *CORO1A*, *MARCKS*, and *MPEG1* (Figures 4A,C), among which *VCAN* showed the most significant change.

### Hub Gene Validation

**FIGURE 5:**
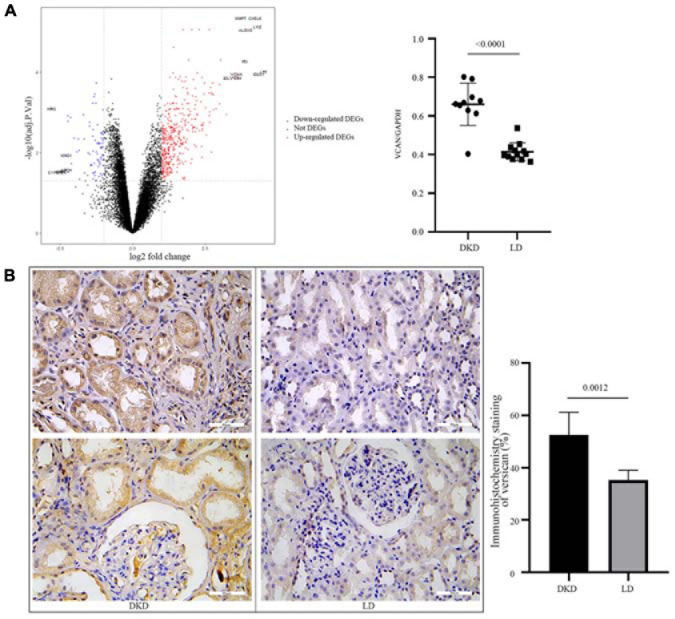
Validation of VCAN in GSE30122. **(A)** Volcano plot of GSE30122 and the top 10 DEGs were annotated. Difference in relative VCAN transcription levels between DKD and LD, *P* < 0.0001. **(B)** Immunohistochemistry staining of versican in DKD and LD kidney tissues.

To further evaluate the consistent change in VCAN in DKD, we performed DEG analysis of GSE30122 (Figure 5A). VCAN was included in the top 10 DEGs. The relative mRNA content of VCAN between DKD and LD was significant (*P* < 0.0001). Additionally, immunohistochemical staining of kidney tissue revealed a higher level of versican expression in the tubulointerstitium from DKD kidney tissue than from LD (Figure 5B).

### Cell Type Enrichment Analysis

**FIGURE 6:**
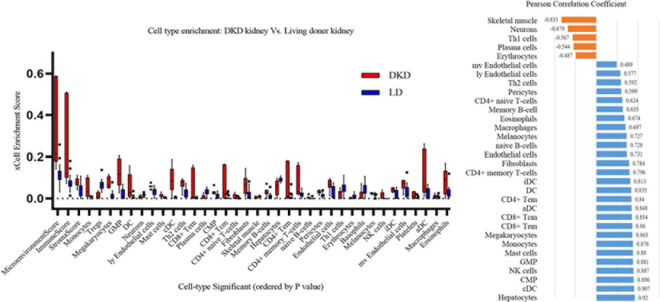
Immune cells enrichment. **(A)** Contrast of cell-type enrichment score between DKD and LD with *P* < 0.05. **(B)** Correlation of versican levels and expression levels ranking of cells in Figure 6A, except for basophils, platelets, and Treg cells which were not correlated with versican expression (*P* > 0.05).

xCell is used for cell type enrichment analysis from gene expression data for 64 immune and stromal cell types. Our data revealed that the DKD group had higher immune scores, microenvironment scores, and stroma scores than the LD group. Of the 64 cell types, scores of 35 types of cells were differentially expressed between DKD and LD, including immune cells, such as monocytes, Tregs, dendritic cells, mast cells, Th2 cells, and CD8^+^ Tem (Figure 6A) cells. Among these 35 cell types, 32 types of cells showed a correlation with *VCAN* expression to varying degrees (Figure 6B).

## Discussion

DKD poses great burdens to the national health system because of its high morbidity and major expenditure. Once it has progressed to the dominant albuminuria stage, DKD continues to progress to end-stage renal disease inexorably. Furthermore, because of the global increase in CKD caused by diabetes mellitus each year, we examined the pathogenesis of DKD through bioinformatics analysis.

To better understand the pathogenesis of DKD, we compared the transcriptome profiles between DKD and LD. Overall, 563 DEGs were identified. According to GO and KEGG analysis, these genes were mostly enriched in immune-related biological processes, such as immune system process, immune response, defense response, and complement and coagulation cascades. These processes may be relevant to various infections and were deduced from enrichment analysis of pathways, such as viral protein interaction with cytokine and cytokine receptor, *Staphylococcus aureus* infection, and pertussis malaria. Most processes occur extracellularly and on the cell surface from adhesion interactions, such as cell adhesion, biological adhesion, signaling receptor binding, cell adhesion molecules, and cytokine-cytokine receptor interaction. Additionally, some vesicle-relevant processes are involved in the progression of DKD, such as the phagosome and extracellular exosome. IPA showed that some immune-relevant pathways were activated, including acute phase response signaling, complement system, and the role of pattern recognition receptors, such as in recognition of bacteria and viruses, leukocyte extravasation signaling, dendritic cell maturation, systemic lupus erythematosus in B cell signaling pathway, interleukin-8 signaling, and the Th1 and Th2 pathways.

The immune response exerted a major influence on the progression of DKD. This result agrees with studies reporting the involvement of immune system components in DKD progression. A wide range of proinflammatory molecules, including cytokines, receptors, chemokines, cell adhesive molecules, and transcription factors, participate in the progression of DKD (Navarro-Gonzalez et al., 2011). Infiltration of a large quantity of immune cells also accelerates the progression; these cells include macrophages, dendritic cells, T lymphocytes, B lymphocytes, neutrophils, and mast cells (Nguyen et al., 2006; Tesch, 2010; Zheng et al., 2012; Yang and Mou, 2017). Thus, immune cell infiltration, proinflammatory cytokines, inflammasome activation, immune complex formation, and complement activation function together to induce kidney injury (Hickey and Martin, 2018). Additionally, some pharmaceuticals developed for DKD targeting inflammatory mediators have been reported as effective to varying degrees (Pichler et al., 2017).

To further examine the relationship between traits and gene expression, we performed WGCNA. The lightcyan module was selected to further study the module with the highest correlation between traits and gene expression. After integrating genes from DEG analysis and WGCNA, we selected several hub genes with high fold-changes and high trait relevance. Among them, VCAN showed the largest difference between the DKD and LD groups.

Versican, translated from the VCAN gene, is a component of the extracellular matrix and plays a role in modulating cell adhesion, proliferation, migration, apoptosis, and ECM assembly. It also functions in inflammation modulation through its interaction with immune cell receptors and chemokines. It exists in four isoforms, namely V0, V1, V2, and V3. These isoforms differ in two alternative splicing glycosaminoglycan domains, which are the chondroitin sulfate (CS) attachment domain (Foulcer et al., 2014). V0, V1, and V3 are found in most tissues. V0 and V1 are major isoforms that accumulate in disease tissues, and it appears that these two isoforms play a role in the inflammation process. V3 is thought to inhibit the pro-inflammatory function of V0/V1 because of its lack of CS domains. V2 is only expressed in the central neuron system (Wight et al., 2014a,b; Wight, 2017). In previous studies of CKD, versican has been discovered as a predictor of disease progression, but there is no study focusing on the influence of versican on DKD tubulointerstitium (Rudnicki et al., 2012; Han et al., 2019; Taylor et al., 2020).

Versican exerts its proinflammatory influence by affecting the adhesion of myeloid and lymphoid cells (Gill et al., 2010; Wight et al., 2014a,b). Adhesion of leukocytes, including activated T-lymphocytes and monocytes, is modulated by versican (Potter-Perigo et al., 2010; Evanko et al., 2012). Moreover, versican participates in extracellular matrix assembly and remodeling. Versican interacts with various other molecules, including hyaluronic acid, tenascin-R, fibulin-1, and fibrillin (LeBaron et al., 1992; Aspberg et al., 1999; Isogai et al., 2002). By controlling extracellular matrix molecule organization, versican modulates cell invasion. Therefore, versican can modulate inflammation cell infiltration in disease tissues. Versican also influences inflammation by modulating cytokine release, such as by inducing the secretion of tumor necrosis factor-α and interleukin-6 in macrophages (Wight et al., 2014a). A pro-inflammatory phenotype is prone to tubulointerstitial remodeling and fibrosis (Hijmans et al., 2017).

We then perform correlation analysis between DEGs and immune cell types as well as between VCAN transcription data and immune cell types. Immune scores in the DKD group were markedly higher than those in the LD group. From another perspective, proinflammatory cells, such as monocytes, dendritic cells, and CD8^+^ Tem (T effector memory) cells increased, whereas anti-inflammation cells, such as Tregs, decreased. Among cells showing differential expression between DKD and LD, the transcription levels of VCAN demonstrated a high correlation with proinflammatory cells, such as classical dendritic cells, natural killer cells, mast cells, monocytes, and CD8^+^ Tem cells. Therefore, versican may reveal inflammation states by indicating immune cell infiltration in DKD. Furthermore, versican may mediate DKD inflammatory injury by influencing the distribution of these immune cells.

There were several limitations to our study. First, our analysis was restricted by the number of samples enrolled, as transcriptome data on DKD tubulointerstitium were limited, and most were tested using different platforms. Therefore, it was difficult to integrate these data. Second, as mentioned above, the transcriptional level of VCAN is related to pro-immune cell expression in DKD tissue; however, it was difficult to draw the conclusion that versican modulated immune cell infiltration, and we will work on these issues in our future studies.

## Conclusion

We identified VCAN as a hub gene in DKD tubulointerstitial injury by integrating DEG analysis and WGCNA. This result was further validated in kidney tissue from patients with DKD and LDs. Moreover, versican was predicted to play a role in immune injury according to the enrichment of functions and signaling pathways. The level of versican was correlated with the assembly of immune cells in the kidney during DKD progression.

## Data Availability Statement

The datasets presented in this study can be found in online repositories. The names of the repository/repositories and accession number(s) can be found below: https://www.ncbi.nlm.nih.gov/, GSE104954; https://www.ncbi.nlm.nih.gov/, GSE30122.

## Ethics Statement

The experiment protocols were approved by the Research Ethics Committee of The First Affiliated Hospital, College of Medicine, Zhejiang University. The patients/participants provided their written informed consent to participate in this study.

## Author Contributions

QX and HJ conceived and designed the project. QX conducted the data analysis and wrote the manuscript. All authors edited and revised the manuscript, and agreed to be accountable for the content of the work.

## Conflict of Interest

The authors declare that the research was conducted in the absence of any commercial or financial relationships that could be construed as a potential conflict of interest.

## Publisher’s Note

All claims expressed in this article are solely those of the authors and do not necessarily represent those of their affiliated organizations, or those of the publisher, the editors and the reviewers. Any product that may be evaluated in this article, or claim that may be made by its manufacturer, is not guaranteed or endorsed by the publisher.

## Footnotes

1
https://www.ncbi.nlm.nih.gov/geo/

2
https://biit.cs.ut.ee/gprofiler/gost

3
https://cran.r-project.org/web/packages/WGCNA/index.html

4
https://xcell.ucsf.edu/

## Abbreviations

CKD: chronic kidney disease
DKD: diabetic kidney disease
LD: living doner
DEG: differentially expressed gene
WGCNA: weighted gene co-expression network analysis
GO: gene ontology
KEGG: Kyoto encyclopedia of genes and genomes
IPA: ingenuity pathway analysis
GS: gene significance
MM: module membership
CS: chondroitin sulfate
Tem cells: T effector memory cells.
