# Supplementary figures and images for: Identification of VCAN as Hub Gene for Diabetic Kidney Disease Immune Injury Using Integrated Bioinformatics Analysis
Source: Front Physiol. 2021 Sep 7;12:651690. doi: 10.3389/fphys.2021.651690 (PMC8454927; doi:10.3389/fphys.2021.651690)

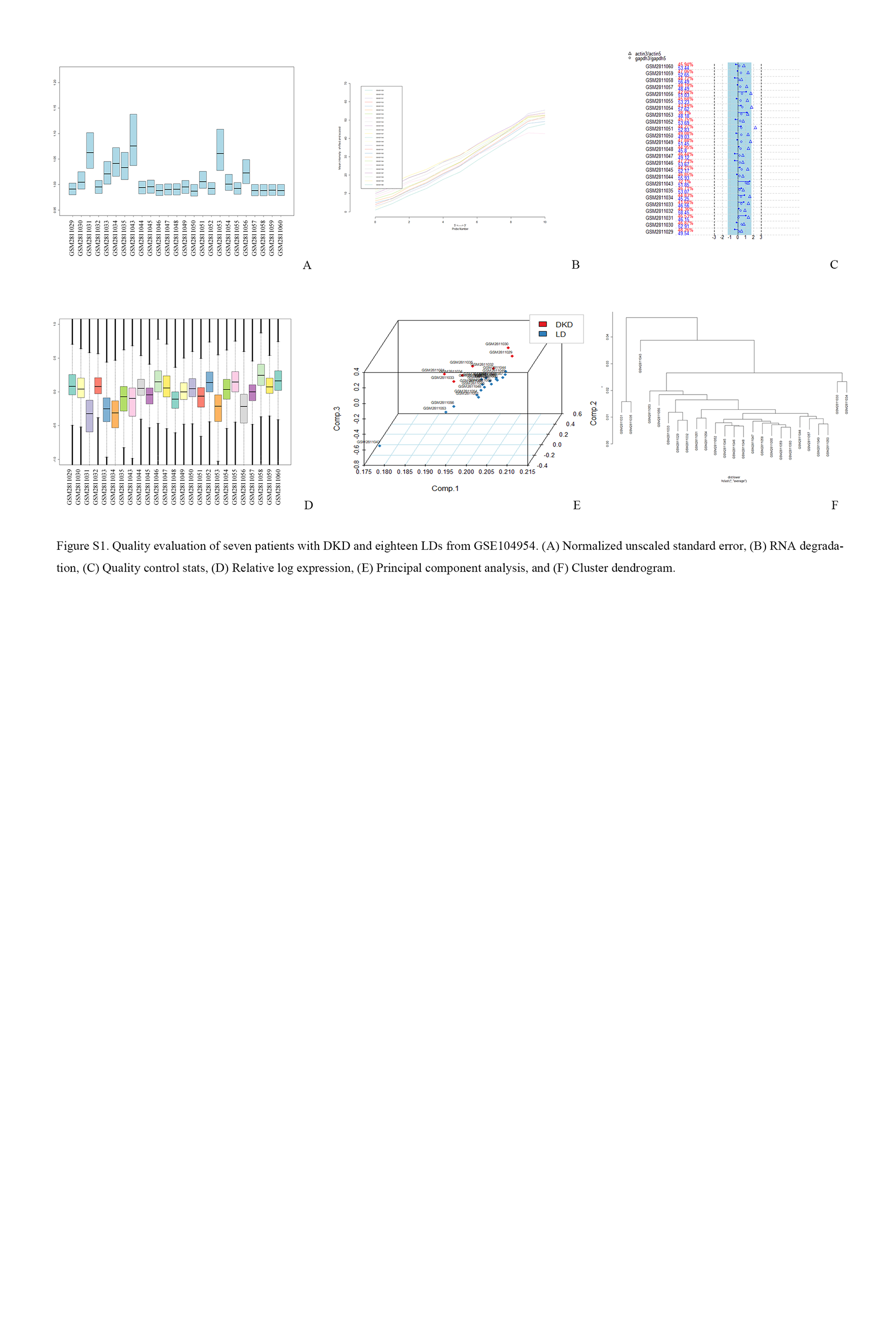

Supplement: Supplementary file 1 [file Image_1.TIF]

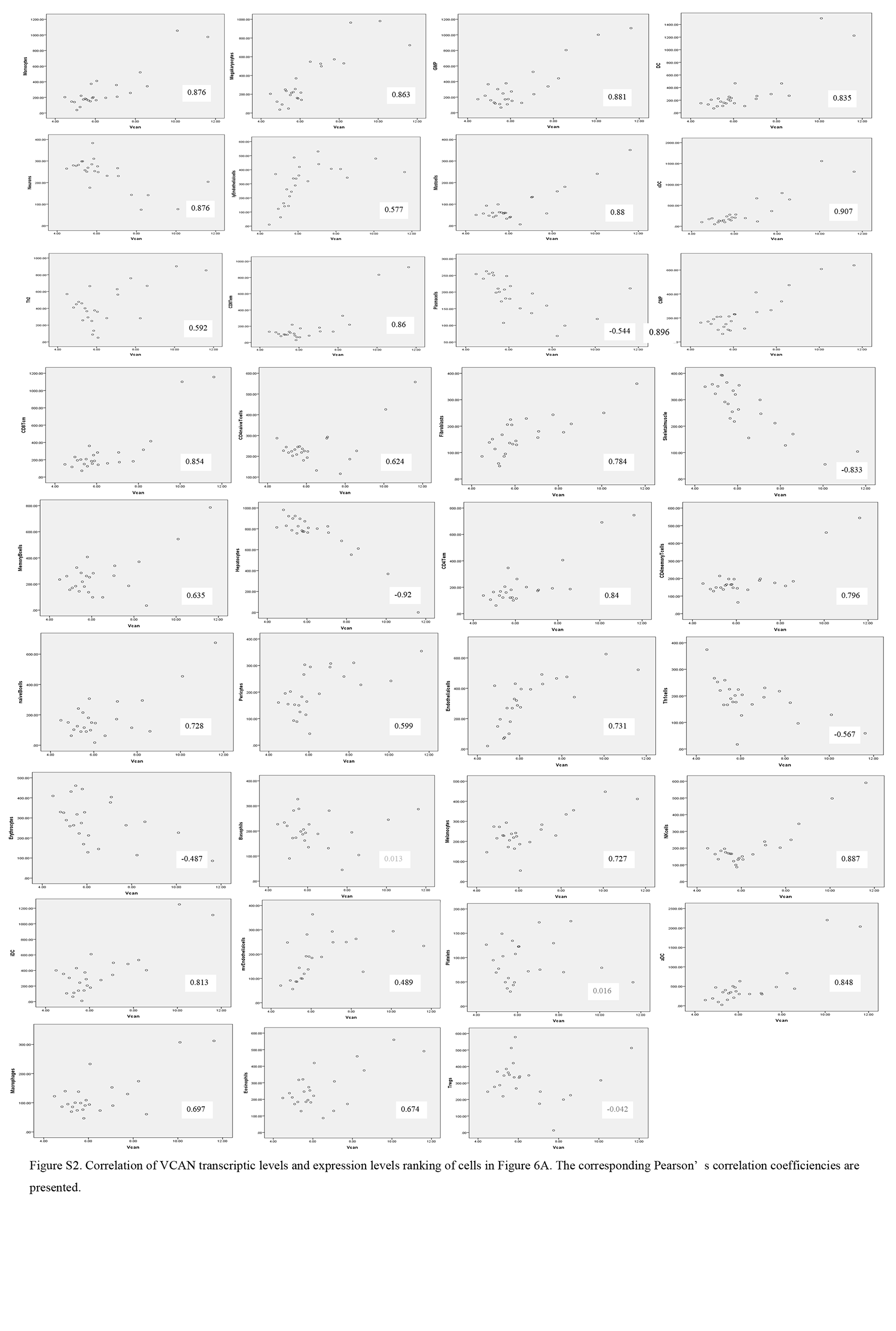

Supplement: Supplementary file 2 [file Image_2.TIF]
