# Supplementary material for: Identification of VCAN as Hub Gene for Diabetic Kidney Disease Immune Injury Using Integrated Bioinformatics Analysis
Source: Front Physiol. 2021 Sep 7;12:651690. doi: 10.3389/fphys.2021.651690 (PMC8454927; doi:10.3389/fphys.2021.651690)
Supplement: Supplementary file 3 [file Table_1.DOCX]

TABLE S1 The 247 downregulated and 316 upregulated differentially expressed genes identified in the gene expression microarray study of seven patients with DKD and seventeen controls.

| **DEGs** | **Gene Symbol** |
| --- | --- |
| Down-regulated | NPHS2 PCOLCE2 BRE-AS1 FGF1 NDNF USP2 CLIC5 PDK4 DUSP1 ALB PSPH ST6GALNAC3 PODXL SIK1 CYP27B1 ZBTB16 ERRFI1 SOST IP6K3 WT1 RP11-999E24.3 CHI3L1 FOS TRPM6 KLK1 PLA2R1 NR4A2 TIPARP FKBP5 TSC22D3 RP11-21L23.2 DPP6 LINC00948 FOSB CLDN11 G6PC PVALB LOC727944 GADD45B NPHS1 TRIM50 CTGF RHCG EGR1 NTNG1 FAM150B APOLD1 FAH CYP3A5 GGACT ATP6V0D2 EGF TMEM178A CISH G0S2 SLC25A25 GADD45A RGS1 CALML3 HRG SLC4A9 SPINK1 SLC25A33 ARHGEF26 C5orf27 SPOCK2 FAM151A ATF3 APOH SOCS2 LINC00473 MAFF LPL SLC12A3 SGK1 FAM24B TCF21 DMRT2 AFM PLCE1 KLF9 PM20D1 RGS3 ETNK2 SLC34A1 KCNE1 SPIDR KLK6 F3 TNNC1 SLC23A3 ZFP36 ATP6V1C2 ADM TNNT2 THY1 GPC3 GATSL3 HTRA1 SPSB1 SLC19A2 NR4A1 LINC00839 NES LINC01187 ATP6V1B1 CEL KLK7 FAM83D SPOCK1 AOC1 APOC3 LOX TSPAN2 METTL21B EGFLAM ELL2 GAD1 FRY TSKU CTTNBP2 TMEM100 SLC22A8 MAPT-AS1 CTSV BAIAP2 SEMA6D PTGDS HEPACAM2 APOD NEBL RGS2 RGCC ZNF331 CSRNP1 NFASC TPPP3 SLC26A7 TMEM52B ETNPPL AGXT PTGER3 STAP1 NR4A3 FABP1 FOXI1 CR1 GPR98 KLHL3 ATP6V1G3 DEFB1 GK3P DNMT3L CYP4A11 MAGI2-AS3 CYP2C8 PPP1R16B SEMA3G SORCS1 MAGI2 XPNPEP2 PRODH2 MACROD1 SLC4A1 HPGD ZFAND5 RNF186 DEPDC7 NXPH2 ANPEP PKP2 MTNR1A DHDH HPD FBXO21 PQLC1 LOC149703 TTC36 RHOB IDNK MRO CIART DDN TMPRSS2 SLC16A7 COL4A3 RALYL AVPR1A LIN52 GLTPD2 LOC644090 KCNH6 GJA3 KLF6 TGFBR3 ARHGAP28 FTCD LOC101929040 SMIM5 DDIT4 GRAMD1B SLC13A2 UPB1 P4HA1 TYRO3 SLCO4A1 GATA6 CDA CTH RGS7 LDHD UNC5D GPR116 SLC31A2 LOC102723845 SLC26A4 LOC439938 SLC51A SH3GL2 ALDH1L1 DPEP1 C15orf59 LDLR NUDT16 TCEAL2 UBE2M GLUD2 FRMD3 NEDD9 RNF152 EAF2 TENM2 IL6R GDF15 KCNK5 AASS STRA6 SLC4A11 DLST EXPH5 CEBPB HAO2 SLC51B ASB9 SLC22A6 VEGFA SLC2A12 |
| Up-regulated | CXCL6 LTF LYZ CTHRC1 VCAN CPA3 FCER1A TAC1 ALOX5 CX3CR1 MMP7 PROM1 IGLC1 REG1A C3 HBB TFPI2 COL1A1 INHBA SLPI RARRES1 CCR2 TPSB2 HOPX IGKC THBS2 VWF TPSAB1 CD52 HS3ST1 PXDN IGLV1-44 TNFAIP8 HAVCR1 SERPINA3 WFDC2 QPCT MPEG1 AGR2 ARL4C SOX4 IGHM TNC CAPN6 CYP24A1 PLK2 CCL19 HTR2B LY96 EVI2A MNDA FAM26F TIMP1 CHODL PTPRC IRX1 CORO1A ACKR1 COL3A1 PLAC8 RAC2 CD44 MOXD1 POSTN GPX8 GIMAP2 FAM129A TNFSF15 PYCARD C16orf54 KLHL14 TNMD TYROBP ANXA1 CLU CSTA HLA-DQB1 CCND2 CLC C15orf48 EVI2B CD48 LTB CDH6 IL27RA RNF144B TGFBI IGDCC4 LY75 COL8A1 GZMA STEAP4 C1S CCL2 TUBA1A CTSS COL15A1 CD53 CMTM3 CSF2RB CP UCP2 FHL2 CASP1 FYB CLDN1 LAMC2 FAM132B MARCKS CXCL1 CRNDE HLA-DPA1 IL10RA MS4A6A OSMR TIFA SAMSN1 CCL21 TESC GCNT3 NLGN4X NKAIN4 LCN2 DOCK11 CCL8 IFIT2 CFB SGK223 TTC39A KLRB1 FN1 IRF8 LAPTM5 IL7 GNG2 PAPPA2 VCAM1 DCDC2 RASSF3 SEL1L3 C1R FLI1 NOV ELK3 C10orf128 PLTP SACS GPM6B RNASE6 SELL TNFRSF11B IRX2 MEST CRIP1 RRM2 PPP1R18 TMSB10 ADAMTS1 CLIC6 LCK PIK3CG GZMK LRRN4 TNFSF13B ANXA3 TLR8 RUNX1T1 CD2 CXCL16 CLEC10A COL6A3 HGF IFI16 TM4SF18 KIAA0101 LOC100132891 SPON1 IL32 GGTA1P ACTN1 NRCAM LUM IGHD C1QA C1RL FIBIN CD1C TSPAN8 VIM GUCY1A3 CHST9 C1QB CCL5 RASSF5 CHST15 LYG1 EMB DYRK2 MACC1 TSPAN13 1-Mar RASSF2 IL7R COL4A1 PSMB9 IGLL5 MCOLN2 ITGAM GPR65 ABCG1 SH3GL3 SAMD9L PTGS2 SFRP2 HDAC9 PHLDA2 TRBC1 TRAC TLR1 APBB1IP LDLRAD4 TPM1 CFD KCNJ8 TGM2 FBN1 MYOF FXYD5 CCDC109B TNFSF10 KDELR3 RAB27B BHLHE41 IRX3 BIRC3 BCL11B FGG MNS1 GPR18 GPR34 PTGFRN CXADR TRIM22 LOC441666 PLSCR1 TMEM54 CDH3 NPY1R FAM198B PPAP2C IFITM1 CARD6 CLEC2B ADAMTS5 CLDN4 AP1S2 SAMHD1 FLRT3 SERPINE2 CYBB RASGRP1 ANXA2 DOCK2 LPCAT1 GIMAP7 CTTNBP2NL CD3D CARD16 CRIP2 RASSF6 SFN RNF182 APOBEC3B GZMB HMHA1 MICAL2 LINC00924 IL33 HRH1 DKK3 TNFAIP2 RGS18 PLP2 GPAM ADAMTSL3 FAM171B STAMBPL1 FPR3 CD36 SLC34A2 BTN3A2 COL14A1 HLA-DMB PTPRE ANXA2P2 KIF26B MST4 IGLJ3 TP53 KRT80 LGALS1 TNFRSF17 LHFP ACKR4 CDH11 GUCY1B3 KDELC1 P2RY13 KCNT2 HPGDS RGS5 TNFRSF19 FCN1 ZBED8 DTX3L PNMA2 |
